# SOLO-SLAM: A Parallel Semantic SLAM Algorithm for Dynamic Scenes

**DOI:** 10.3390/s22186977

**Published:** 2022-09-15

**Authors:** Liuxin Sun, Junyu Wei, Shaojing Su, Peng Wu

**Affiliations:** College of Intelligence Science and Technology, National University of Defense Technology, Changsha 410073, China

**Keywords:** SLAM, SOLO-SLAM, SOLO_V2, deep learning, navigation, robotics

## Abstract

Simultaneous localization and mapping (SLAM) is a core technology for mobile robots working in unknown environments. Most existing SLAM techniques can achieve good localization accuracy in static scenes, as they are designed based on the assumption that unknown scenes are rigid. However, real-world environments are dynamic, resulting in poor performance of SLAM algorithms. Thus, to optimize the performance of SLAM techniques, we propose a new parallel processing system, named SOLO-SLAM, based on the existing ORB-SLAM3 algorithm. By improving the semantic threads and designing a new dynamic point filtering strategy, SOLO-SLAM completes the tasks of semantic and SLAM threads in parallel, thereby effectively improving the real-time performance of SLAM systems. Additionally, we further enhance the filtering effect for dynamic points using a combination of regional dynamic degree and geometric constraints. The designed system adds a new semantic constraint based on semantic attributes of map points, which solves, to some extent, the problem of fewer optimization constraints caused by dynamic information filtering. Using the publicly available TUM dataset, SOLO-SLAM is compared with other state-of-the-art schemes. Our algorithm outperforms ORB-SLAM3 in accuracy (maximum improvement is 97.16%) and achieves better results than Dyna-SLAM with respect to time efficiency (maximum improvement is 90.07%).

## 1. Introduction

Simultaneous localization and mapping (SLAM) is one of the core technologies in the field of robotics. Based on slam technology, mobile robots can realize their real-time localization and environment map construction through sensor data in unknown environments. Owing to the wide range of promising applications in autonomous driving, robotics, and virtual reality technologies, visual SLAM techniques have received increasing attention from researchers in recent years. After long-term research, the current visual slam system has formed a basic framework composed of visual odometry, state estimation backend, map construction, and loopback detection [1]. Based on the above classical framework, several SLAM algorithms have been proposed, such as ORB-SLAM3 [2], VINS-Fusion [3], DSO [4], etc. Several of these algorithms have excellent performance and can already meet the application requirements in most cases. The theoretical framework of the SLAM system has become relatively complete. However, as the application scenarios become increasingly complex, the SLAM system also shows obvious shortcomings in some specific real-world scenarios. For example, when the robot runs in an indoor scene, it will inevitably face the situation of people walking. In the classical SLAM framework, both the feature point method and the direct method rely on the strong assumptions of rigid scenarios. This assumption limits the application of SLAM algorithms in real-world environments. The features extracted by the SLAM system from moving objects (such as pedestrians) will lead to incorrect data association results. This will greatly interfere with the visual localization and map building processes [5].

In order to solve this problem, researchers have gradually formed three improvement directions [6]: (1). a filtering method based on the conventional model. Researchers filter out outliers based on traditional methods such as RANSAC, optical flow [7], and epipolar constraints. (2). A filtering method based on the deep learning model. The SLAM system is based on the use of a deep learning segmentation algorithm to obtain the a priori dynamic regions in the image. After that, the feature points within the priori dynamic region are filtered out. (3). An enhancement method based on the deep learning model. Researchers further enhance the SLAM system based on the semantic information obtained by the second method. Since the deep learning-based method usually has a better filtering effect, the system can usually achieve better localization accuracy. In contrast, the segmentation task of deep learning will consume a great deal of time. Taking Dyna-SLAM [8], a classical open-source solution based on the second improvement idea, as an example, the system needs to complete the segmentation task to get the semantic region before it can further perform the bit pose estimation in the tracking thread of the SLAM system. This also leads to the overall processing time being the sum of the time consumed by the segmentation task and the processing time of the SLAM system, and the overall real-time performance is poor.

Aiming at the problem of positioning accuracy and real-time operation in indoor dynamic scenes, this paper proposes a new SLAM algorithm (SOLO-SLAM), which combines the above three improvement ideas. The algorithm performs two rounds of dynamic point filtering design by combining instance segmentation and geometric constraints. This greatly enhances the localization accuracy of the system in dynamic scenes. Distinct from algorithms such as Dyna-SLAM, SOLO-SLAM will update the dynamic state and semantic attributes of mapped points in a separate semantic thread. By design, SOLO-SLAM can perform filtering operations without waiting for the semantic segmentation results of the current frame. This greatly enhances the real-time performance of the system. In addition, SOLO-SLAM also designs a new constraint based on the semantic information of the environment and the semantic attributes of map points to improve the constraint reduction problem caused by dynamic point filtering. The main contributions of our approach are summarized as follows:

1. We design a SLAM system (SOLO-SLAM) in which the segmentation and pose estimation tasks are fully processed in parallel. SOLO-SLAM updates the dynamic state and semantic attributes of map points in the semantic thread. When processing the current frame, the system can perform the first round of filtering according to the dynamic state of map points without waiting for the semantic segmentation result of the current frame. 

2. We propose an auxiliary filtering method based on geometric constraints combined with the dynamic ranking of regions. The method performs different levels of filtering strategies for different regions based on geometric constraints by dividing the dynamic degree of the regions.

3. We introduce a new semantic constraint based on the semantic attributes of map points. The method performs a new constraint construction based on the relationship between the semantic attributes of map points and the semantic region of the current frame.

The remainder of this paper is structured as follows. Section 2 describes related work. Section 3 outlines our proposed algorithm. Section 4 presents the experimental results in detail. Section 5 discusses our results and summarizes the main conclusions.

## 2. Related Works

At present, the basic design idea of the SLAM system for dynamic scenes is to filter the information introduced by moving objects as outliers. Based on this design idea, the general work can be divided into (1) filtering methods based on the traditional model and (2) filtering methods based on the deep learning model. Our proposed SOLO-SALM system adopts a two-round filtering strategy. In the first round of filtering, it is based on the deep learning method. In the second round of filtering, it is based on the traditional geometric model approach. We present the current related excellent work in Section 2.1 and Section 2.2, respectively.

With the development of deep learning, researchers are increasingly trying to use the semantic information of the environment obtained from deep learning to enhance the performance of the system. In the filtering process of the SOLO-SLAM system, we can get the semantic information of the environment. Therefore, we tried to design a new semantic constraint to enhance the performance of the system by using the semantic information. We introduce relevant work in Section 2.3.

### 2.1. Filtering Method Based on Conventional Model

Before the widespread use of deep learning, researchers used traditional models for filtering. The related work is as follows. Kim et al. [9] proposed the background model-based dense-visual-odometry (BaMVO) algorithm for RGB-D sensors. This algorithm computes a nonparametric background model to represent the background information in the scene based on the acquired scene depth. Then, the anomalous motion information in the scene is extracted and filtered by means of background-based subtraction. Sun et al. [10] used particle filtering and applied the maximum a posteriori (MAP) method for vector quantized images to enhance the boundary information of moving objects obtained based on the temporal difference technique. However, the computational overload of the algorithm and the requirement that the static region must be the main region somewhat limit the practical value of the algorithm. Li et al. [11] assigned static weights to the depth edge points obtained based on depth discontinuities. They proposed a static weighting method for depth edge points in key frames to assist in reducing the impact caused by dynamic information. Mur-Artal et al. [12] utilized the classical outlier filtering algorithm RANSAC [13] in ORB-SLAM2 to filter the more representative feature points as much as possible, thus reducing the impact caused by anomalous data. Pire et al. [14] used a robust cost function in PTAM to reduce the impact of introduced outliers on pose optimization.

### 2.2. Filtering Method Based on Deep Learning Model

With the development of deep learning (DL), numerous breakthroughs have been achieved with respect to object detection and segmentation, especially for dynamic information filtering. DL-based segmentation algorithms can identify a priori dynamic regions in an image. After that, the feature points within the a priori dynamic regions are filtered. Compared to conventional models, depth methods can usually filter out dynamic information better. Therefore, systems based on depth models can usually achieve better localization accuracy. 

To achieve precise localization and map building, B. Bescos et al. [8] proposed Dyna-SLAM based on ORB-SLAM2, with the incorporation of dynamic object detection. The filtering of dynamic objects involves two processes: obtaining semantic segmentation results for dynamic regions in the scene based on the MaskRcnn algorithm [15] and implementing the region growth algorithm to grow regions around dynamic pixels in the image. By combining the two strategies, Dyna-SLAM achieves almost complete filtering of the key points in dynamic regions. Nevertheless, Dyna-SLAM, a classical open-source DL-based approach, needs to complete the segmentation task of identifying the semantic region before it can further perform the task of pose estimation and tracking, leading to poor real-time performance. Yu et al. [16] proposed DS-SLAM by introducing a semantic segmentation approach that combines a semantic segmentation network with a moving consistency check method to reduce the influence of dynamic objects, thus improving localization accuracy in dynamic environments. Additionally, DS-SLAM can generate dense semantic octree graphs for advanced tasks such as navigation. Furthermore, PSP-SLAM, proposed by Long et al. [17], incorporates the idea of semantic segmentation. By utilizing the reverse ant colony strategy, the system avoids dynamic and static judgments for all feature points, reducing processing time during detection. All above-mentioned DL-based algorithms have achieved better localization accuracy compared to the traditional methods. However, they show poor real-time performance due to the substantial amount of time consumed by DL methods. In their Detect-SLAM framework, Zhong et al. [18] employed a DL-based algorithm (i.e., single shot multi-box object detector (SSD)) to detect a priori dynamic objects in the environment. When SSD detects an a priori dynamic object, it removes all feature points in the object region and simultaneously updates the dynamic information of the map points. Subsequently, the system uses dynamic information to assist in feature point selection. Although Detect-SLAM is also based on the sequential processing of detecting a priori regions and then filtering them, it updates the dynamic information of map points to assist in feature selection. We were partly inspired by this in our work. Similarly, Liu et al. [19] developed RDS-SLAM, in which the dynamic probability of map points is updated based on a semantic segmentation algorithm to assist the system in dynamic point filtering. However, both of these schemes have certain problems that are illustrated in more detail in the next section. We also verify the effectiveness of our algorithm by comparing its performance with that of Detect-SLAM and RDS-SLAM in Section 4.

### 2.3. Enhancement Method Based on Deep Learning Model

Several studies have explored the utilization of obtained environmental information (e.g., semantic information) to improve system performance. E. Sucar et al. [20] proposed a monocular scale correction algorithm based on semantic information. This algorithm first acquires the boundaries of vehicles based on a target detection algorithm (YOLO). Then, the actual height of the vehicle is obtained by projecting the 3D points in the local map onto a 2D image to determine the 3D points corresponding to the boundary points of the vehicle area. Finally, scale correction is performed based on the actual and a priori height of the vehicle. Zhang et al. [21] used PWC-Net [22] to obtain the corresponding optical flow residuals of RGB images to highlight the dynamic regions in RGBD point clouds, thereby enabling self-motion estimation and static semantic background reconstruction excluding dynamic information. To enhance optimization, Lianos et al. [23] constructed a new cost function based on the distance relationship between feature points with vehicle semantic attributes and the vehicle mask area in the image. Our semantic optimization improvements are inspired by related ideas. Based on this, we propose a semantic optimization approach supporting up to 80 semantic categories for indoor scenes. Simultaneously, we adopt a new idea for constructing cost functions. Additionally, our constraint construction approach is more reasonable compared to VSO.

## 3. Materials and Methods

In this section, we introduce SOLO-SLAM from three aspects. First, we outline the overall framework of SOLO-SLAM. The main contents of the overall framework are 1. the improvements over ORB-SLAM3 and 2. the advantages of improving the real-time performance of the system compared to common algorithms incorporating semantic segmentation. Afterwards, we introduce the filtering algorithm based on the dynamic degree of the region. This method combines semantic information and geometric constraints to filter anomalous data introduced by dynamic map points. Finally, we develop an optimization strategy based on semantic information of indoor scenes. The method is implemented based on the semantic attributes of map points and the semantic regions on pictures obtained from instance segmentation.

### 3.1. Overview of SOLO-SLAM System

**Figure 1:**
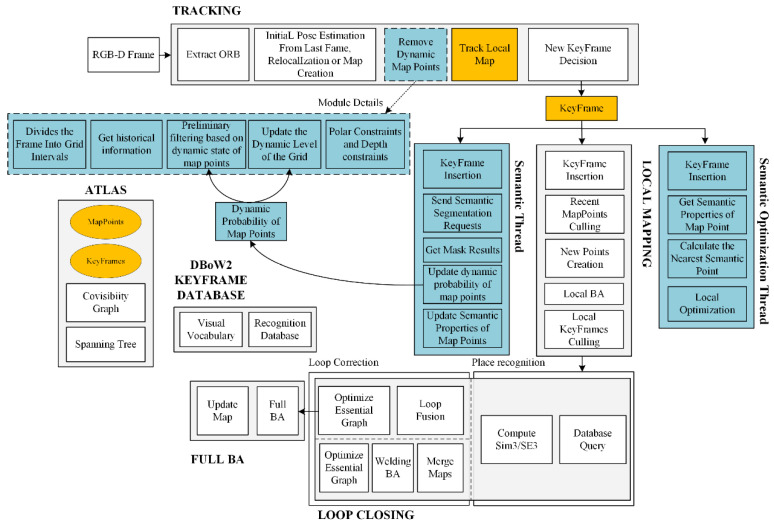
Overall structure of the SOLO-SLAM system (blue area is the new content added compared to ORB-SLAM3; the dashed box offers detailed description of the module; and the orange area presents the content modified).

The SOLO-SLAM system aims to improve the performance of ORB-SLAM3. It includes five threads to process tasks. As shown in Figure 1, ORB-SLAM3 consists of three main threads: tracking, local mapping, and loop closing. Based on these threads, ORB-SLAM3 shows excellent performance in static scenarios. For the necessity of program implementation, we improved the orange part in Figure 1. The storage of instance segmentation results was added to the KeyFrame. Semantic attributes, dynamic states, and modifications related to nearest neighboring points were added to MapPoints. The track local map module added new support for dynamic information processing. Specific applications are explained step by step with the new contents below.

We added three main parts on top of the above. As shown in the blue part of Figure 1, we first added a new semantic thread. This thread operates on key frames. Its functions are as follows.

The semantic thread is responsible for saving the newly created key frames and sending segmentation requests to the SOLO-V2 [24] instance segmentation function package based on the Robot Operating System (ROS). After obtaining the instance segmentation results, the dynamic probability and semantic attributes of map points in the system are updated in conjunction with the mask of the key frames. We describe this in detail from a real-time perspective in Section 3.2.

Inside the original tracking thread of ORB-SLAM3, we added a new filtering module for dynamic features. The details are as follows.

**Figure 2:**
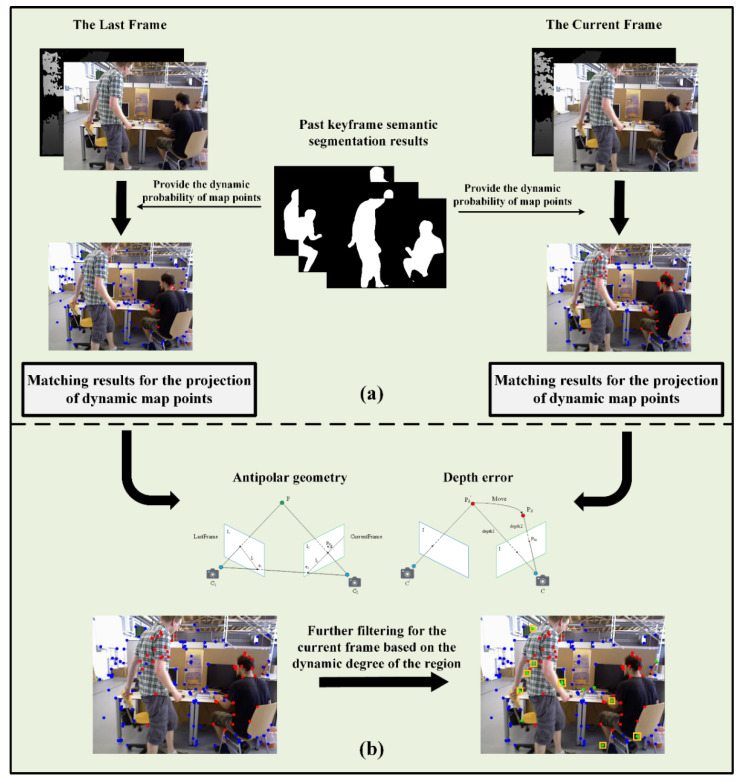
The dynamic point filtering method of SOLO-SLAM. (**a**) corresponds to the first round of filtering. (**b**) corresponds to the second round of filtering. The red points represent the map point projection identified as dynamic points in the first round of filtering. The blue points represent the map point projection identified as static points in the first round of filtering. The green points selected in the yellow rectangular box correspond to the points filtered in the second round.

Our overall design of the dynamic feature filtering module is shown in Figure 2. The module is divided into two parts: (a) and (b). Figure 2a illustrates the preliminary filtering method based on the dynamic probability of map points. The basic idea is to update the dynamic probability of map points in the semantic thread based on the segmentation result of the a priori dynamic region. Consequently, the dynamic state of the map point is updated based on the dynamic probability. Finally, all the projection points corresponding to the dynamic map points are filtered in the tracking thread. It can be seen that there are still some blue points in the region corresponding to the human. Therefore, we perform a second round of filtering. As shown in Figure 2b, the operation can be divided into three steps: 1. computing the dynamic degree of the region, 2. filtering using an epipolar constraint, and 3. re-judgment based on the depth error. We introduce the method for the second round of filtering in detail in Section 3.3.

In addition, we added a semantic optimization thread for key frame operations, with the following function.

The semantic optimization thread is responsible for saving the key frames processed by the semantic thread. Additionally, it is responsible for building new semantic constraints based on the relationship between semantic attributes and semantic masks of map points. The semantic optimization method is combined with the optimization method based on the re-projection error. Relevant implementation details are described in Section 3.4.

The above new semantic threads and semantic optimization threads run completely in parallel with other threads, thus fully guaranteeing the independence of the tracking threads. We describe the above three improvements below.

### 3.2. Real-Time Oriented Dynamic Information Filtering Method 

#### 3.2.1. Introduction to the Parallel Processing Design

**Figure 3:**
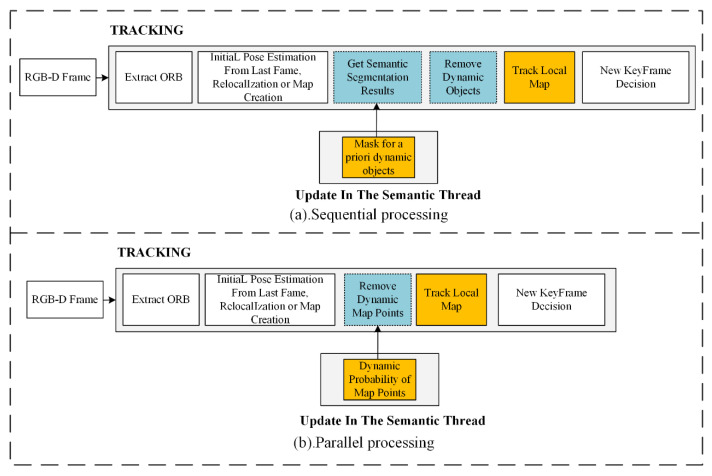
Comparison of sequential and parallel processing structures.

Usually, after training DL models, we can obtain the location of a priori dynamic targets in the scene based on semantic segmentation methods. Therefore, in recent years, researchers have developed numerous DL-based methods for identifying dynamic target regions and consequently filtering dynamic features in visual odometry. However, these methods suffer from serious real-time problems. Figure 3a shows that in most existing methods, dynamic region filtering is added to the tracking thread. Then, an additional semantic thread is created to acquire the a priori dynamic region. Although the two threads are running in parallel from the thread perspective, the tracking task is not really processed in parallel. Based on this idea, the actual workflow is as follows:

Step 1: Wait for the semantic thread to obtain the segmentation result of the current frame in the tracking thread.

Step 2: Filter all feature points in the a priori dynamic target mask area in the tracking thread.

Step 3: Compute the pose in the tracking thread based on the filtered projection points.

In this approach, the semantic thread only plays the role of sending semantic segmentation requests, while the tracking thread must wait for the segmentation task to be completed before filtering the dynamic feature points. Therefore, although the above method adds a semantic thread, in fact, the calculation of pose still needs to wait for the completion of the segmentation task.

Since the semantic segmentation method takes a long time, whereas feature extraction often takes very little time, the actual time consumed by the tracking thread is the sum of the semantic segmentation time ($t_{sem}$), the dynamic feature filtering processing time ($t_{\mathrm{process}}$), and the pose estimation time ($t_{track}$). 

Among the three, the semantic segmentation time is usually much longer than the sum of the latter two. Therefore, to a certain extent, even if we integrate the task of sending semantic segmentation requests in the semantic thread into the tracking thread, it will not have too much impact on the whole system time. In this article, we call this method sequential processing. In contrast, our proposed SOLO-SLAM enables parallel processing of the instance segmentation task and the pose calculation task.

Our proposed SOLO-SLAM is based on the motion model for tracking after initialization is completed. The motion model [2] is implemented as follows:

Step 1: The pose transformation result obtained from the previous frame of the system is used as the initial value.

Step 2: Project map points to the current frame for matching.

Step 3: According to the matching results, graph optimization is performed based on the re-projection error to obtain the pose of the current frame.

As shown in Figure 2a, sequential processing filters all the projection points in the a priori dynamic region based on the obtained mask. When tracking based on the motion model, our projection points are derived from the projection of map points. In essence, filtering the projection points in the a priori dynamic target mask can be transformed into identifying the dynamic map points. When the map points are dynamic, filtering can be achieved without using the corresponding projection points.

Therefore, we specifically design the parallel processing method shown in Figure 2b. Compared to the sequential processing approach, the pose calculation for the current frame can be performed directly without waiting for the instance segmentation task. The workflow is as follows:

Step 1: Filter the feature points matched by the dynamic map points in the current frame in the tracking thread.

Step 2: Calculate the pose based on the filtered feature points in the tracking thread.

The difference between our method and the sequential processing method illustrated in Figure 1 is that we do not need the semantic segmentation results for each frame. Therefore, the SLAM system can reduce the semantic segmentation time $t_{sem}$ in the tracking thread.

(1) $$\begin{array}{l} t_{\mathrm{total}\_sp}=t_{sem}+t_{process}+t_{track}\\ t_{\mathrm{total}\_pp}=t_{process}+t_{track} \end{array}$$

In the above parallel processing steps, when it is determined that the map points are dynamic points, the corresponding projection matching results are filtered. The dynamic state of map points is determined by the dynamic probability of map points. Therefore, the problem is further transformed into the updating of the map point probability in the semantic thread.

#### 3.2.2. Dynamic State Update Implementation of Map Points

As shown in Algorithm 1, we show the specific process of the dynamic probability update in the semantic thread. Next, we present the details of the dynamic probability update and dynamic status update of map points.

**Dynamic probability update:** In the semantic thread, we only perform instance segmentation on representative frames (Key frames) and update the dynamic probability of map points according to the a priori dynamic target mask and the location relationship of projection points. The specific dynamic probability update strategy [19] is as follows:

(2) $$\begin{array}{l} \{\begin{array}{l} p_{d\_\mathrm{temp}\;}=\varepsilon_{d}\cdot p_{d\_\mathrm{last}\;}\\ p_{s\_\mathrm{temp}\;}=\varepsilon_{s}\cdot p_{s\_\mathrm{last}\;} \end{array}\\ \varepsilon =\frac{1}{p_{d\_temp}+p_{s\_temp}}\\ \left\{ \begin{array}{l} p_{d\_\mathrm{current}\;}=\varepsilon \cdot p_{d\_temp}\\ p_{s\_\mathrm{current}\;}=\varepsilon \cdot p_{s\_temp} \end{array}\right. \end{array}$$

where $p_{d\_last}$ and $p_{s\_last}$ are the dynamic and static probability of the last update, respectively. $p_{d\_\mathrm{current}\;}$ and $p_{s\_\mathrm{current}\;}$ are the dynamic and static probability, respectively, of the current key frame after the update based on instance segmentation. ε is the normalization coefficient. p is the update factor. We set p to 0.7. The sum of $\varepsilon_{d}$ and $\varepsilon_{s}$ is 1. When the projection point is in the a priori dynamic region, $\varepsilon_{d}$ is equal to p; when the projection point is outside the a priori dynamic region, $\varepsilon_{s}$ is equal to p.

In the updating process of dynamic probability, the algorithm involves the projection of map points. The projection here refers to the projection of a point in the world coordinate system to the pixel coordinate system. The relevant formula is as follows:

(3) $$P_{uv}=\left[\begin{array}{c} u\\ v\\ 1 \end{array}\right]=\frac{1}{Z}\left(\begin{array}{ccc} f_{x} & 0 & c_{x}\\ 0 & f_{y} & c_{y}\\ 0 & 0 & 1 \end{array}\right)\left(\begin{array}{c} X\\ Y\\ Z \end{array}\right)\overset{\;\mathrm{def}\;}{=}\frac{1}{Z}KP=\frac{1}{Z}KTP_{w}$$

where $P_{uv}$ corresponds to the projection point on the pixel coordinate system. P corresponds to a point on the camera coordinate system. $P_{w}$ corresponds to the map point on the world coordinate system. K corresponds to the internal parameter matrix of the camera. T corresponds to the transformation matrix from the world coordinate system to the camera coordinate system.

**Figure 4:**
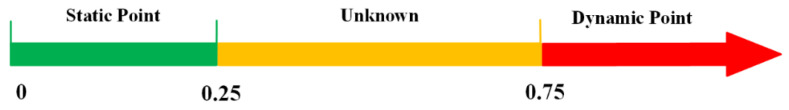
Correspondence between dynamic states of map points and dynamic probabilities.

**Dynamic status update:** As shown in Figure 4, we consider a map point as dynamic when its dynamic probability is greater than 0.75. In the tracking thread, we perform a preliminary filtering operation on the projection matching results of dynamic map points.

$M^{\mathrm{in}\_\mathrm{view}}$ is the collection of all map points with matching relationships within the field of view of the current key frame to be optimized. $M^{project}$ is the set of projection positions of $M^{\mathrm{in}\_\mathrm{view}}$ on the current key frame. $m^{\mathrm{sem}}$ and $m^{dynamic}$ are member variables of map points, corresponding to semantic attribute probability and dynamic probability, respectively. $R^{\mathrm{sem}}$ is the collection of all semantic regions of the current key frame. $r^{dynamic}$ and $r^{static}$ are the a priori dynamic and the a priori static regions of the current key frame, respectively. $KF^{sem}$ is the set of key frames awaiting instance segmentation.

As shown in Equation (1), the time consumed by the SLAM system for pose estimation completely excludes the effect of the instance segmentation consumption time by the design of parallel processing. However, the update of the dynamic probability of map points depends entirely on the instance segmentation results of key frames, which poses the following two problems.

1. The map points detected by key frames do not cover all map points generated by normal frames. Therefore, the map point probability update is not comprehensive.
2. The processing speed of normal frames in the tracking thread is higher than that of key frames in the semantic thread. Therefore, there is a certain lag in updating the probability of map points.

Compared to the sequential execution method, the above two shortcomings make it difficult to completely filter the projection points from dynamic targets during normal frame processing. Therefore, according to the characteristics of parallel processing methods, we introduce a secondary filtering method based on regional dynamic degree and geometric constraints in Section 3.3.
**Algorithm 1** Update dynamic probability and semantic properties of map points$\begin{array}{l} \mathrm{Input}:\;M^{\mathrm{in}\_\mathrm{view}}\;KF^{optimize}\\ \mathrm{Output}:\;M^{project}\;R^{sem}\\ \mathrm{For}\;kf^{optimize}\;in\;KF^{optimize}\;\mathrm{do}:\\ \;\;//\mathrm{Get}\;\mathrm{instance}\;\mathrm{segmentation}\;\mathrm{results}\;\mathrm{based}\;\mathrm{on}\;\mathrm{ROS}\\ \;\;R^{sem}\leftarrow RequestInstanceSegmentation(kf^{optimize});\\ \;\;r^{dynamic}\leftarrow GetDynamicRegion(R^{sem});\\ \;\;r^{static}\leftarrow GetStaticRegion(R^{sem});\\ \;\;M^{project}\leftarrow GetProjectionofMappoints\;(M^{\mathrm{in}\_\mathrm{view}});\\ \;\;//\mathrm{Update}\;\mathrm{only}\;\mathrm{the}\;\mathrm{semantic}\;\mathrm{attributes}\;\mathrm{of}\;\mathrm{map}\;\mathrm{points}\;\\ \;\;//\mathrm{whose}\;\mathrm{projections}\;\mathrm{lie}\;\mathrm{in}\;\mathrm{static}\;\mathrm{regions}\\ \;\;\mathrm{For}\;m^{project}\;in\;M^{project}\;\mathrm{do}:\\ \;\;\;\;m^{dynamic}\leftarrow UpdateDynamicProbability\;(m^{project},r^{dynamic});\\ \;\;\;\;\;\mathrm{If}\;(m^{project}\;\mathrm{in}\;r^{static})\;\mathrm{then}\\ \;\;\;\;\;\;m^{sem}\leftarrow UpdateSemanticProperty(m^{project},r^{static});\\ \;\;\;\;\mathrm{End}\;\mathrm{If}\\ \;\;\mathrm{End}\;\mathrm{For}\\ \mathrm{End}\;\mathrm{For} \end{array}$

### 3.3. Filtering Method Based on the Dynamic Degree of the Region

#### 3.3.1. Regional Dynamic Degree Acquisition

**Figure 5:**
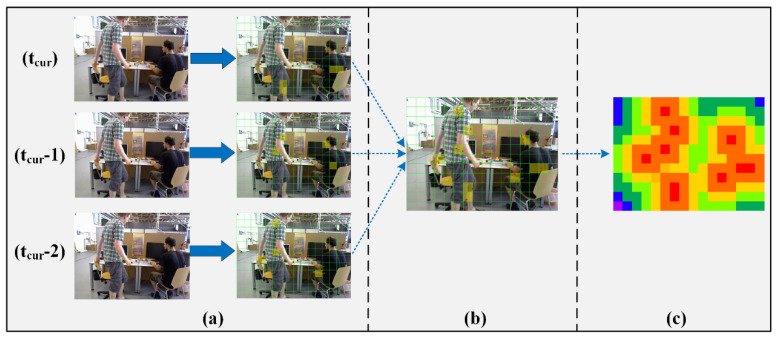
Regional dynamic degree acquisition. (**a**) corresponds to the dynamic point projection distribution of the last three frames. (**b**) corresponds to the projection distribution of dynamic points after merging the last three frames. (**c**) corresponds to the regional dynamic degree of the current frame.

As shown in Figure 5a, we divide the image region into m*n grid regions, inspired by the quadtree approach, to assign feature points. According to the dynamic state of the map points, we can detect the projection points for some dynamic map points. The red points in the image are the corresponding projection points. We label the grid area where the dynamic map points are projected as the dynamic area. The yellow grid area in the image corresponds to the dynamic area. As analyzed above, the dynamic status update of map points is lagging and incomplete. Therefore, when the current frame is divided into dynamic regions, sometimes a problem arises in that there are too few dynamic regions. Owing to the high frame rate of the camera, the a priori dynamic target displacement between adjacent frames is usually small. Therefore, in the short term, the historical dynamic region has a strong reference. In the SOLO-SLAM system, we save the dynamic regions of the first two frames and fuse them with the current frame. As shown in Figure 5b, the fused dynamic regions have richer information.

The a priori dynamic target usually corresponds to a whole region. The grid region closer to the dynamic region is more likely to be part of the region where the a priori dynamic target is located. Therefore, we divide the dynamic degree of the grid region based on the distance from the dynamic grid region. As shown in Figure 5c, the dynamic regions are highlighted in red color. As the distance increases, it gradually changes to a cooler color. By dividing the grid area, the computational effort can be greatly reduced, and the robustness is enhanced.

After obtaining the dynamic degree of the regions based on distances, we need to convert the distances into proportions. The proportions are used for the threshold division of different regions in the subsequent operation. The conversion is performed using the sigmoid logistic regression function. The formula is

(4) $$thr_{scale}=\frac{1}{1+e^{-\delta \cdot d}}$$

where d is the distance from the dynamic grid region. δ indicates the transformation coefficient. Thereby, we can obtain information about the scale of the distance and use it for the threshold setting.

#### 3.3.2. Outlier Determination Based on the Epipolar Constraint

**Figure 6:**
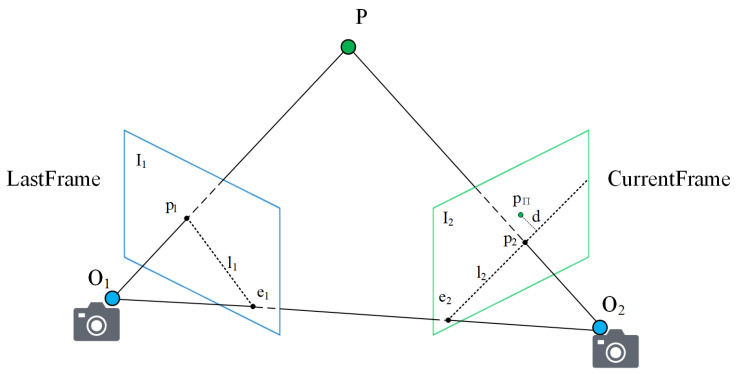
The epipolar constraint.

As shown in Figure 6, $I_{1}$ and $I_{2}$ are RGB images obtained by a camera at the previous and current moments. $O_{1}$ and $O_{2}$ are the corresponding optical center positions of the camera at the previous and current moments, respectively. P is a map point that is jointly observed under adjacent moments. Theoretically, the projection of a map point at the current moment will be on a ray consisting of poles $e_{2}$ and $p_{2}$ if the attitude is absolutely accurate. However, owing to the motion of dynamic objects, the projection of dynamic map points as anomalous data does not satisfy the above constraint. Moreover, the projection corresponding to the dynamic map point is usually at a farther distance from the ray $\vec{e_{2}p_{2}}$ compared to normal data. The projection point ($p_{\Pi }$) is shown in the image. Once we obtain the dynamic degree of the region, we can further filter outliers based on the distance between the projection point and the ray.

After the initialization is completed, we perform tracking based on the motion model approach. The pose obtained from the calculation of the last frame is used as the initial value of the pose. Therefore, it is not necessary to calculate the fundamental matrix to solve for the initial value of the pose. Instead, the construction of the epipolar constraint relies on the fundamental matrix. Therefore, to perform the filtering operation in combination with estimating the distance and the dynamic degree for the regions, our work is divided into two steps.

1. Obtain the basis matrix: We first perform a preliminary matching based on Harris corner detection. Subsequently, the matching results are further optimized based on the optical flow pyramid. We also filter the points with large luminance differences in the 3 ∗ 3 grid region [16] around the corner point locations. Finally, we calculate the most representative fundamental matrix F with the RANSAC outlier filtering algorithm.
2. Obtain the distance information: the distance here refers to the distance of each projection point from the ray ($\vec{e_{2}p_{2}}$) at the current frame. After obtaining the fundamental matrix, we use the following equation to compute the feature points in Figure 6.

(5) $$\begin{array}{l} p_{1}=\left[u_{p_{1}},v_{p_{1}}\right]\\ p_{\pi }=\left[u_{p_{\pi }},\nu_{p_{\pi }}\right] \end{array}$$

where u and v correspond to the image pixel coordinates.

The homogeneous coordinates of $p_{1}$ and $p_{\pi }$ are expressed as

(6) $$\begin{array}{l} p_{1}^{\prime }=\left[u_{p_{1}},v_{p_{1}},1\right]\\ p_{\pi }^{\prime }=\left[u_{p_{\pi }},\nu_{p_{\pi }},1\right] \end{array}$$

where $l_{2}$ is $\vec{e_{2}p_{2}}$ in Figure 6. Theoretically, the projection point should fall on $l_{2}$. F is the fundamental matrix between adjacent frames.

(7) $$l_{2}=\left[\begin{array}{l} X_{2}\\ Y_{2}\\ Z_{2} \end{array}\right]=F\cdot {\left(p_{\pi }^{\prime }\right)}^{T}=F\left[\begin{array}{c} u_{p_{\pi }}\\ v_{p_{\pi }}\\ 1 \end{array}\right]$$

Based on the formula for the distance from a point to a line, we can obtain

(8) $$\mathrm{dis}\left(p_{\pi },l_{2}\right)=\frac{\left|p_{\pi }^{\prime }\cdot F{\left(p_{1}^{\prime }\right)}^{⊤}\right|}{\sqrt{{\left\|X_{2}\right\|}^{2}+{\left\|Y_{2}\right\|}^{2}}}$$

By iterating through the projection points of all map points in the current frame, we can calculate the distances for all projection points. After arranging the distances from small to large, we can get the distance set $V_{dis}=\left\{dis_{1},dis_{2}\ldots dis_{n}\right\}$. Each map point projection selects the filtering threshold in $V_{dis}$ in proportion according to $thr_{scale}$ obtained through Equation (4). When the distance corresponding to the map point is greater than the threshold and the corresponding state is static, it is preliminarily determined that the current static map point is a dynamic map point that has not been updated. The corresponding projection points are added to the set $V_{outlier}$ for filtering decisions based on depth information.

#### 3.3.3. Outlier Determination Based on Depth Error

**Figure 7:**
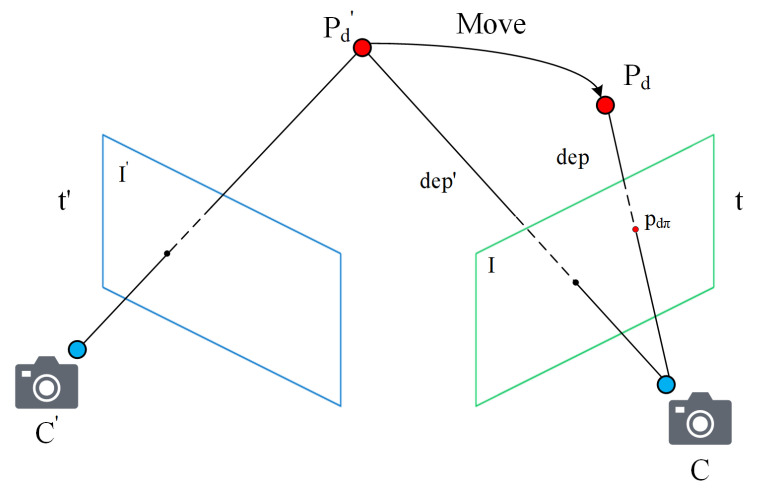
Depth error.

For tracking, we can obtain the position of the camera in the world coordinate system at each time. Simultaneously, the system continuously generates map points that have world coordinates. Theoretically, there is fixed distance information between any two known points in 3D space. However, as shown in Figure 7, at a certain historical time $t^{\prime }$, the camera captures the image $I^{\prime }$. Simultaneously, the system generates a map point ${P_{d}}^{\prime }$. With the continuous movement of the camera, at the current time t, the camera captures the image I. If ${P_{d}}^{\prime }$ is a dynamic map point, ${P_{d}}^{\prime }$ will move in the time interval from $t^{\prime }$ to t. The feature points on our matching are actually the projection points $p_{d\pi }$ corresponding to the map points that have moved to $P_{d}$.

Since RGB-D cameras can provide RGB images and depth images simultaneously, we can use the depth image obtained at the current time to determine the corresponding motion state of map points. Based on the pixel coordinates of the projected points, we can look up the corresponding pixel positions on the depth image to estimate the corresponding depth values dep. According to the 3D coordinates of the map point $P_{d}{}^{\prime }$ generated in the past and the camera position of the previous frame, we can calculate the distance $dep^{\prime }$ between the two points. The formula is expressed as follows.

(9) $$\begin{array}{l} dep^{\prime }=\sqrt{{\left(P_{cw}-P_{d}{}^{\prime }\right)}^{2}}\\ dep=\mathrm{dep}(P_{d\pi }) \end{array}$$

where $P_{cw}$ is the coordinates of the camera at the previous frame. $P_{d}{}^{\prime }$ is the coordinates of the map point itself. During the operation of the SLAM system, both $P_{cw}$ and $P_{d}{}^{\prime }$ are known. We only need to calculate the distance between the two points. $P_{d\pi }$ is the projection matching point. dep(x) refers to the search operation at the corresponding pixel coordinates in the depth image. We can get dep by finding the depth value at the pixel coordinates corresponding to $P_{d\pi }$ on the depth images.

Then, we calculate the ratio of the current depth to the error.

(10) $$dep_{err}=\frac{dep}{\left|dep-{dep}^{\prime }\right|}$$

**Figure 8:**
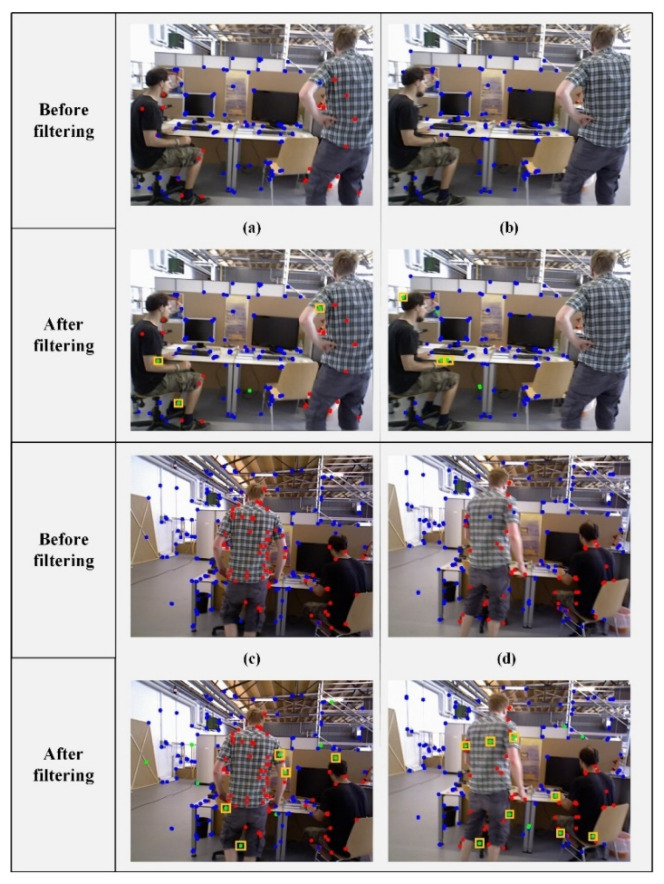
Further comparison of filtering performance. In the figure, (**a**,**b**) are two adjacent frames. (**c**,**d**) are randomly extracted frames. Red points represent the projections of dynamic map points. Blue points represent the projections of static map points. Green points are the projections of filtered map points in the second round. The points selected in the yellow rectangular box correspond to the green points in the a priori dynamic region.

By traversing all the matching points in $V_{outlier}$, we obtain the corresponding $dep_{err}$ and set the empirical threshold $dep_{thr}$. To improve the accuracy of dynamic scenes, dynamic feature points should be filtered and effective matching should be retained as much as possible to construct constraints. Therefore, when $dep_{err}$ is greater than $dep_{thr}$, we remove the corresponding matching point from $V_{outlier}$. After two rounds of filtering determination, the matching points in $V_{outlier}$ are treated as dynamic points in the pose estimation for the current frame. Thereby, we can achieve better filtering performance. Figure 8 presents a direct comparison of filtering performance. Figure 8a,b are pictures at are two adjacent frames. We can see that (b) presents an extreme case. Owing to the lag in the dynamic information update of map points, there is no reference dynamic map point projection in (b). Through the division of regional dynamic degrees based on historical information, we further filter the projection points of some a priori dynamic regions through two rounds of employing constraints.

### 3.4. Constraint Construction Based on Semantic Information

We have added semantic threads and semantic optimization threads to SOLO-SLAM. The semantic thread is responsible for updating the dynamic probability and semantic attributes of the map points based on instance segmentation. After obtaining the semantic attributes of the map points and the semantic masks of the key frames, we combine them in the semantic optimization thread to build a new optimization method. Next, we describe the specific implementation details.

#### 3.4.1. Implementation Details of the Semantic Threads

**Figure 9:**
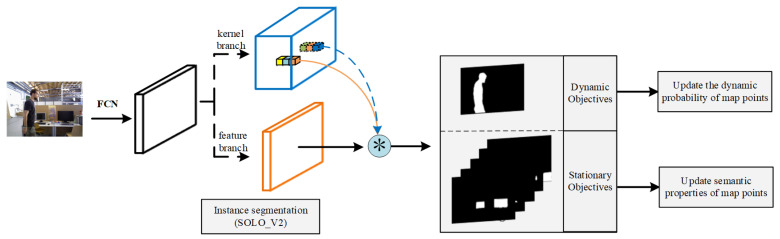
Processing operations in semantic threads.

**Instance segmentation method:** In the SOLO-SLAM system, we perform mask acquisition using a DL algorithm after creating key frames. As shown in Figure 9, to obtain the semantic information of the image, we use the state-of-the-art instance segmentation algorithm (SOLO_V2) to perform the image segmentation task. The core idea of SOLO_V2 is to transform the segmentation problem into a positional classification problem. It innovatively decouples the original mask into a mask kernel branch and a mask feature branch, which are used to predict the convolutional kernel and convolutional feature, respectively. After improvement, SOLO_V2 shows excellent performance. Details about SOLO_V2’s usage are as follows:

- SOLO-SLAM is designed based on the ROS system. We designed the ROS interface program for SOLO_V2. The data interaction with the SLAM part is realized by encapsulating SOLO_V2 as a function package.
- The SOLO_V2 algorithm is trained on the COCO dataset [25] and can recognize up to 80 categories. The recognized objects include people, bicycles, cars, and other potential motion objects. The plentiful recognizable categories can help us to recognize both common a priori dynamic objects and environmental information such as TVs, chairs, and bottles in the scene. This can provide sufficient support for our semantic constraint construction.
- The SOLO-SLAM system runs on the PyTorch DL framework. The application of SOLO_V2 algorithm is implemented with the help of MMdection Python toolbox.
- The instance segmentation algorithm in SOLO-SLAM uses a 50-layer backbone network (solov2_light_512_dcn_r50_fpn_8gpu_3x.py) provided by the authors of SOLO-V2. The training and use involved in the SOLO-SLAM system are implemented based on this network structure.

**Mask usage details:** After obtaining the instance results via SOLO-V2, SOLO-SLAM first updates the probability of map points based on the projected points in the a priori dynamic region. After the dynamic probability update is completed, the mask of the a priori static region is traversed, and the semantic attributes of the map points are updated according to the mask class of the region where the projection points are located.

(11) $$\left\{ \begin{array}{l} p_{sem1\_\mathrm{current}\;}=[\varepsilon_{sem}\cdot p_{sem1\_last}+(1-\varepsilon_{sem})]p_{flag}\\ p_{sem2\_\mathrm{current}\;}=[\varepsilon_{sem}\cdot p_{sem2\_last}+(1-\varepsilon_{sem})]p_{flag}\\ \cdots \cdots \\ p_{semn\_\mathrm{current}\;}=[\varepsilon_{sem}\cdot p_{semn\_last}+(1-\varepsilon_{sem})]p_{flag} \end{array}\right.$$

where $p_{semn\_\mathrm{current}\;}$ is the probability corresponding to the nth semantic attribute of the current map point. We take the semantic attribute with the largest probability value as the semantic attribute of the current map point. $\varepsilon_{sem}$ is the update factor, which can be set to any value less than 0.5 based on experience. When the map point projection falls in the corresponding semantic region, $p_{flag}$ is set to 1. Otherwise, $p_{flag}$ is set to 0. In contrast to the dynamic probability update method, when the projection corresponding to the map point is in the nth new semantic region for the first time, we set $p_{semn\_\mathrm{current}\;}$ equal to 0.5. Afterwards, the update is performed according to Equation (9). We maintain the update of only two semantic attributes in SOLO_SLAM. When it falls in the third semantic region, we consider that the semantic attribute of the map point has extremely low confidence. Consequently, the corresponding map point will no longer participate in semantic optimization.

#### 3.4.2. Implementation Details of the Semantic Optimization Thread

**Figure 10:**
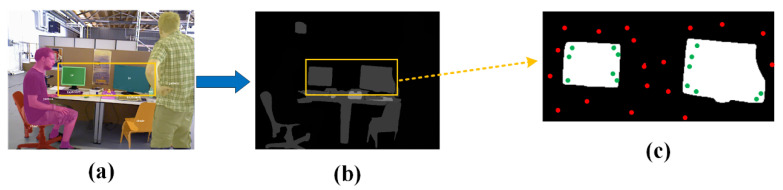
Semantic constraint principle. (**a**) shows the result of the Instance segmentation. (**b**) shows the result after being converted into a grayscale image. (**c**) is a schematic diagram of projection points.

**Semantic constraint principle:** Objects in real-world environments have semantic information. The ORB corner points [26] detected from the objects by the SLAM system also have semantic properties corresponding to the objects. As shown in Figure 10a, the instance segmentation algorithm allows us to assign semantic labels to objects in the environment. Combining the semantic labels of objects and the semantic attributes of map points, we can impose constraints. Taking the monitor as an example, theoretically, when the pose is absolutely accurate, the projections of map points from the monitor at the current frame should necessarily be inside the semantic region corresponding to the monitor. However, owing to pose errors, the projections of map points are often scattered near the semantic region as shown in Figure 10c. Therefore, it can be used as a constraint that the projections of map points with semantic attributes should be inside the region with the same semantic labels.

**Construction of semantic constraints:** SLAM systems based on feature point methods usually use the minimization of the re-projection error to impose constraints. In the tracking process, we can obtain the projections of map points in the current frame and the corresponding matching points. Consequently, the pose and map points can be continuously optimized by methods such as graph optimization or extended Kalman filtering. The paper uses a graph optimization method, i.e., a nonlinear optimization method. The code implementation is based on the g2o [27] functional package. In this paper, the graph optimization method is designed to use the 3D coordinates of map points obtained during the operation of the system and the pose at each moment as optimization vertices respectively. At the same time, constraints are constructed as edges connecting vertices. Our main work lies on the design of new constraints. The quantitative index in the optimization process is the distance of the projection point from the matching point. By minimizing the distance, the SLAM system can improve the system accuracy. Similarly, we transform the semantic constraint into the error between 2D coordinates and minimize the distance as a quantitative indicator. Two constraints are represented as follows:

(12) $$\begin{array}{l} err_{re\_proj}=K\cdot T_{cw}\cdot P-p_{\mathrm{match}}\\ err_{sem\_proj}=K\cdot T_{cw}\cdot P-p_{nearest} \end{array}$$

where $err_{re\_proj}$ is the constraint constructed based on the re-projection error. $err_{sem\_proj}$ is the constraint constructed based on semantic information. $T_{cw}$ is the transformation matrix. P represents the map points. $p_{\mathrm{match}}$ represents the matching points. As we can see, the key is the acquisition of $p_{nearest}$.

In the semantic thread, we save the static region mask corresponding to each key frame. The acquisition of the mask is dependent on the mask information. The relevant process can be divided into four steps as follows.

Step 1: Iterate through the mask categories of all the a priori static regions and calculate the center of mass for the mask regions. Since instance segmentation can distinguish between different individuals of the same category, the search area can be further refined.

Step 2: Determine whether there is more than one mask region in the same category. When there are multiple mask regions, the mask with the closest distance to the projection point is selected as the target mask.

Step 3: Evaluate the position relationship with the semantic region according to the projected position.

Step 4: Extract the target region contour. Search for the nearest neighboring point on the contour as $p_{nearest}$.

In SOLO-SLAM, we perform graph optimization based on g2o. While delineating edges, we update the position relations in step 3 in real time. When an object is inside the semantic region, we temporarily remove the corresponding edge in this optimization round. The overall optimization is performed simultaneously with the re-projection error-based optimization. The cost function at the final optimization is defined as

(13) $$T=\arg\min\sum_{i}err_{re\_proj}(k,i)+\lambda \sum_{i}err_{sem\_proj}(k,i)$$

where T is the pose to be optimized, k is the index of the key frame, i is the index of all map points in the current key frame, and λ is the weighting factor. We set λ to 0.3. Using the above method, we introduce more constraints to facilitate optimization. As shown in Algorithm 2, we also provide pseudocode to demonstrate the process. In the next section, we compare our system with other state-of-the-art algorithms to demonstrate its overall performance.
**Algorithm 2** Update Nearst Point of Map Point$\begin{array}{l} \mathrm{Input}:\;M^{\mathrm{in}\_\mathrm{view}}\;M^{project}\;R^{sem}\\ \mathrm{Output}:\;M^{\mathrm{in}\_\mathrm{view}}\\ \mathrm{For}\;kf^{optimize}\;in\;KF^{optimize}\;\mathrm{do}:\\ \;\;{\mathrm{For}\;m}^{\mathrm{in}\_\mathrm{view}}\;in\;M^{\mathrm{in}\_\mathrm{view}}\;\mathrm{do}:\\ \;\;\;\;m^{project}\leftarrow GetProjectionPoints\;(M^{project});\\ \;\;\;\;m^{\mathrm{sem}}\leftarrow GetSemanticProperties(m^{\mathrm{in}\_\mathrm{view}});\\ \;\;\;\;//\mathrm{Iterate}\;\mathrm{over}\;\mathrm{the}\;\mathrm{semantic}\;\mathrm{region}\;\mathrm{of}\;\mathrm{the}\;\mathrm{current}\;\mathrm{frame}\;\\ \;\;\;\;//\mathrm{to}\;\mathrm{get}\;\mathrm{the}\;\mathrm{nearest}\;\mathrm{point}\\ {\;\;\;\;\mathrm{For}\;r}^{\mathrm{sem}}\;in{\;R}^{\mathrm{sem}}\;\mathrm{do}:\\ \;\;\;\;\;\;sem\_temp\leftarrow \;GetRegionSemanticProperties(r^{\mathrm{sem}});\\ \;\;\;\;\;\;{\mathrm{If}(m}^{\mathrm{sem}}==sem\_temp)\;\mathrm{then}\\ \;\;\;\;\;\;\;\;p^{temp}=GetNearstPo\mathrm{int}ofContours(m^{project});\\ \;\;\;\;\;\;\;\;p^{nearst}\leftarrow UpdateNearstPoint(m^{\mathrm{in}\_\mathrm{view}}->p^{nearst},p^{\mathrm{temp}});\\ \;\;\;\;\;\;\mathrm{End}\;\mathrm{If}\\ \;\;\;\;\mathrm{End}\;\mathrm{For}\\ \;\;\mathrm{End}\;\mathrm{For}\\ \;\;\mathrm{SemanticOptimizationViaG}2O(M^{\mathrm{in}\_\mathrm{view}},M^{project});\\ \mathrm{End}\;\mathrm{For} \end{array}$
where $M^{\mathrm{in}\_\mathrm{view}}$ is the set of all map points with matching relationships within the field of view of the current key frame to be optimized. $M^{project}$ is the set of projected positions of $M^{\mathrm{in}\_\mathrm{view}}$ on the current key frame. $R^{\mathrm{sem}}$ is the set of all semantic regions of the current key frame. $m^{\mathrm{sem}}$ and $p^{nearst}$ are member variables of map points. $KF^{optimize}$ is the set of key frames awaiting semantic optimization.

## 4. Experimental Results and Discussion

In this section, we demonstrate the effectiveness of our algorithm by conducting experimental tests on a publicly available dataset. We compare our SLAM system with other state-of-the-art SLAM systems in terms of accuracy and real-time performance.

### 4.1. Dataset and Algorithms for Comparison

Our proposed SOLO-SLAM system is an RGBD image-based SLAM system for dynamic scenes. Therefore, we select the public TUM dynamic objects dataset [28] for evaluation. The associated dataset was produced based on a kinect camera. Since the kinect camera is based on TOF technology for depth acquisition, we can get a series of 640 ∗ 480 color and depth images. The corresponding datasets are divided into two categories based on the dynamic degree of scenes: w, which stands for walking, corresponding to a highly dynamic scene; and s, which stands for sitting, corresponding to a low dynamic scene. The specific scene is two people sitting at a table, chatting and gesturing. Based on the type of camera motion, the dataset is divided into four categories: (1) static: the sensor is kept in place manually; (2) xyz: the sensor moves along three directions (xyz) while keeping the same orientation; (3) halfspare: the sensor moves on a small half sphere of approximately one meter diameter; and (4) rpy: the sensor rotates along the principal axes (roll–pitch–yaw) at the same position. We provide the comparative results of SOLO-SLAM with other algorithms on the above dataset below.

In the selection of algorithms for comparison, on the one hand, ORB-SLAM3 is considered the most advanced algorithm for static scenes at present; on the other hand, our SOLO-SLAM system is based on ORB-SLAM3 and aims at improving its performance. Therefore, we first selected ORB-SLAM3 as one of the comparison algorithms. In addition, we also selected four SLAM algorithms for comparison with respect to dynamic scenes. Among them, Dyna-SLAM and DS-SLAM are two well-known advanced open-source SLAM systems. In Section 2, we discussed Detect-SLAM and RDS-SLAM from the perspective of applying the dynamic probability of map points, so we also included them in the comparison to demonstrate the effectiveness of our proposed algorithm.

### 4.2. Tracking Accuracy Evaluation

The absolute trajectory error (ATE) corresponds to the difference between the true value of the camera pose and the estimated value of the SLAM system. This criterion is well suited to evaluating the performance of visual SLAM systems. Table 1 and Table 2 show the ATE results of the algorithm under different scenarios. We used the accuracy data provided in the relevant studies as comparison data. For the data not provided in the Dyna-SLAM and DS-SLAM studies, we ran the open-source code and selected the mean values after multiple tests for comparison. For unavailable data, we used "-" to indicate them.

The root mean square error (RMSE) and standard deviation (SD) values are reported in the tables to better demonstrate the robustness and stability of the system. The mean is the mean value of errors in the table, which can better reflect the accuracy information of the system. Referring to the information in the tables, we can see that the accuracy of SOLO-SLAM is improved by one order of magnitude compared to ORB-SLAM3 on the highly dynamic dataset. Among them, the mean and RMSE show the highest improvements of 97.46% and 97.63%, respectively, in highly dynamic scenarios, and 37.84% and 43.55%, respectively, in low dynamic scenarios. This fully validates the effectiveness of SOLO-SLAM in improving system accuracy and stability in highly dynamic scenes. Additionally, this proves the rationality of the idea of parallel processing based on the dynamic state of map points. Compared to the results of the original studies of Detect-SLAM, RDS-SLAM, and DS-SLAM, our algorithm achieves a significant accuracy improvement on almost all datasets tested.

This is most likely due to two reasons. First, the secondary filtering method based on the dynamic degree of the region mitigates the problem of untimely and insufficient dynamic state updates of map points. Second, the SOLO-V2-based instance segmentation mask can obtain more accurate semantic information compared to the target detection frame obtained from SSD. Unfortunately, although the accuracy of the SOLO-SLAM system has been greatly improved, there is still a gap between it and Dyna-SLAM under highly dynamic scenarios. This is because Dyna-SLAM is a sequential processing structure that can fully acquire the a priori dynamic regions in each frame. Dyna-SLAM achieves the best dynamic point filtering performance by combining segmentation results and the region growth algorithm. In contrast, the dynamic point filtering capability of SOLO-SLAM’s parallel processing approach is limited. However, it is worth noting that the SOLO-SLAM system has the highest accuracy in low dynamic scenes compared to all algorithms tested in this study. This is because the ORB-SLAM3 mechanism based on RANSAC and co-view can filter some of the anomalous dynamic points in low dynamic scenes to a certain extent. Nevertheless, the direct filtering of the a priori dynamic region feature points will result in losing many feature points in low dynamic scenes, leading to fewer constraints that can be constructed during local optimization. In contrast, the SOLO-SLAM system adds constraints to the optimization process based on the semantic attributes of map points, which improves the accuracy in low dynamic scenes to a certain extent.

**Figure 11:**
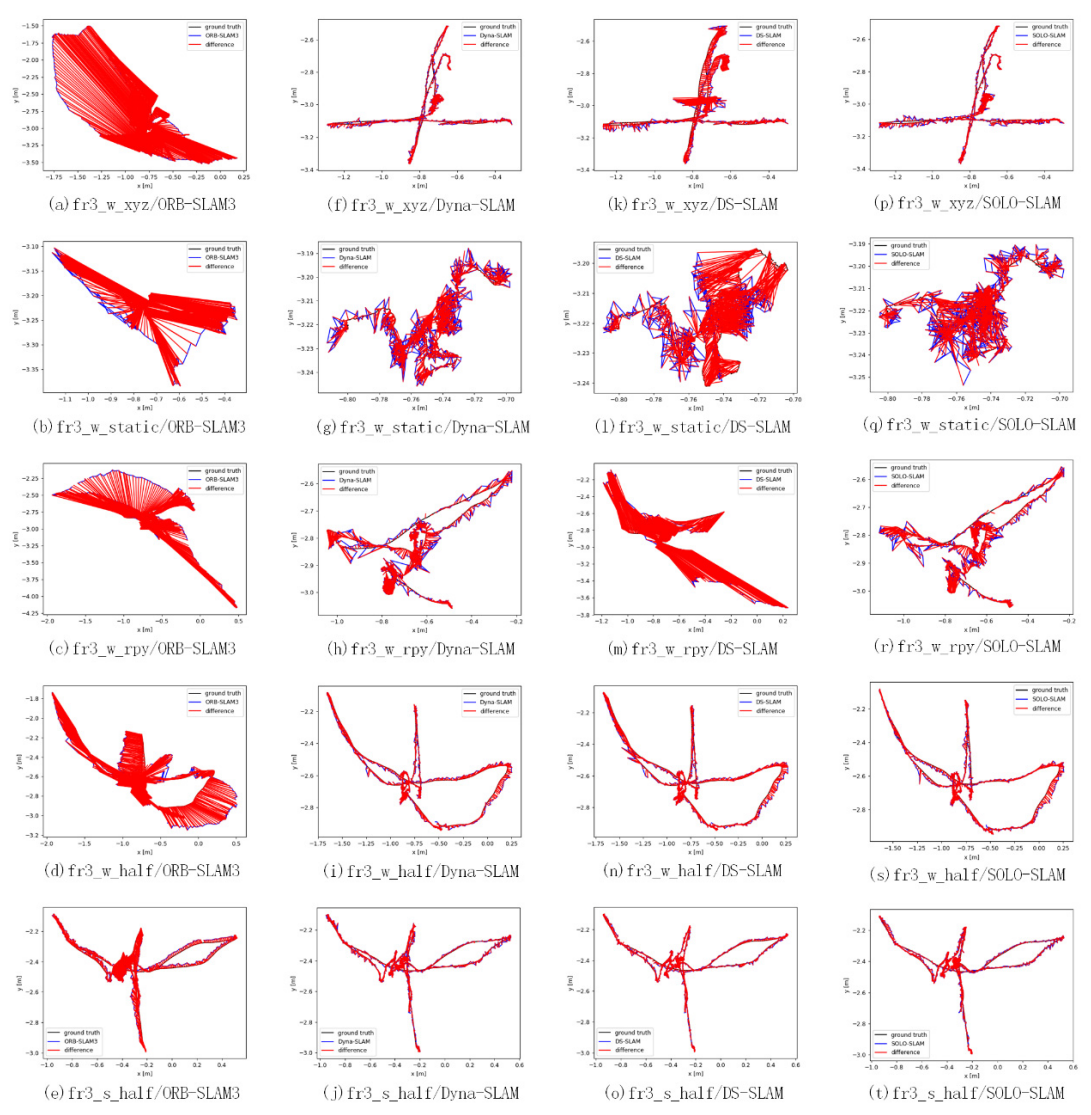
Track diagrams of different dynamic scenes. ORB-SLAM3 corresponds to (**a**–**e**); Dyna-SLAM corresponds to (**f**–**j**); DS-SLAM corresponds to (**k**–**o**); SOLO-SLAM corresponds to (**p**–**t**). The red line corresponds to the difference between the ground truth and the calculated trajectory.

The relative pose error (RPE) is the difference in the amount of positional change within the same two time stamps. This criterion is suitable for estimating the drift of the system. Table 3 and Table 4 show the comparison results in terms of both translations and rotations, respectively. The increments between the relative positions used to calculate the errors in the table are set to 0.1 m. The results are similar to the ATE results. It is worth noting that sequences based on pure rotational motion (rpy) have a relatively low lifting effect. This is probably due to the presence of a large number of 45° rotations of the sensor, which lead to a more difficult update of the dynamic state and semantic attributes of the map points. Compared to methods such as Dyna-SLAM with sequential execution, some dynamic feature points are not effectively eliminated. We also provide estimation results of different algorithms and the ground truth in Figure 11 to provide intuitive comparison.

### 4.3. Real-Time Evaluation

In terms of equipment, both SOLO-SLAM and Dyna-SLAM were tested using a laptop computer. The CPU of the computer was an Intel-i7-12700H with an NVIDIA-3070TI graphics card. Owing to the cuDNN version required for DS-SLAM configuration, we ran the algorithm with an NVIDIA-1060 graphics card. Therefore, we only compared the real-time performance of Dyna-SLAM and SOLO-SLAM on the same device.

SOLO-SLAM adopts a parallel processing approach. Theoretically, the time consumed by SOLO-SLAM is completely independent of the time consumed by instance segmentation. However, the fast processing of images by the tracking threads may result in significant lag in updating the dynamic state and semantic attributes of map points, which will further affect the accuracy of the system. Therefore, the results of our tests were controlled for using a processing speed of 15 frames per second. The implementation was conducted by adding a time delay between frames.

**Figure 12:**
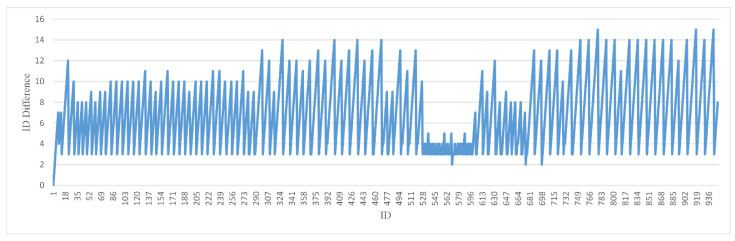
The difference between the nearest keyframe ID and the current frame ID.

**Figure 13:**
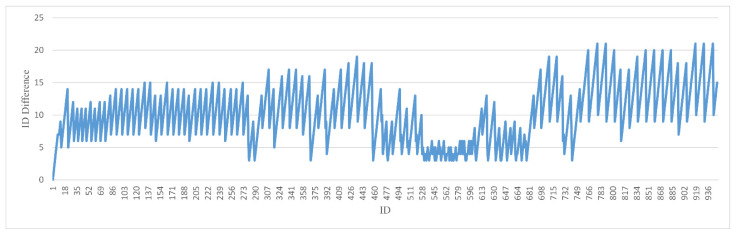
The difference between the oldest keyframe ID and the current frame ID.

We provide the difference between the current frame ID being processed in the test process and the key frame ID that had just completed the instance segmentation. We hope that Figure 12 and Figure 13 can give other researchers an intuitive understanding of hysteresis. When performing instance segmentation on key frames that have not yet been instance segmented, we take turns to select the most recently obtained key frame and the earliest key frame for segmentation. Therefore, we can see that there are two corresponding graphs. Figure 12 corresponds to the minimum difference in processing each frame with an average lag of 6.8 frames Figure 13 corresponds to the maximum difference in processing each frame with an average lag of 10.2 frames.

Table 5 shows the real-time improvement effect of SOLO-SLAM’s parallel processing approach compared to Dyna-SLAM. Combining 3.2 and 3.3, we can see that our algorithm achieves better accuracy improvement compared to sequential processing methods, such as DS-SLAM. Further, compared to Dyna-SLAM, SOLO-SLAM achieves better accuracy in low dynamic scenes. Although there is still a slight difference in accuracy in high dynamic scenes, SOLO-SLAM is still of practical importance due to the substantial improvement in real-time performance.

### 4.4. Evaluation in Real Environment

In order to further prove the effectiveness of the algorithm, we carried out experimental verification using a realsense d435 camera in real scenes. The resolution of the camera is 640 * 480. The acquisition frame rate of the camera is set to 30 frames per second. Relying on the realsense d435 camera, we can obtain the RGB image and depth image of the scene.

Our verification is divided into two parts.

**Figure 14:**
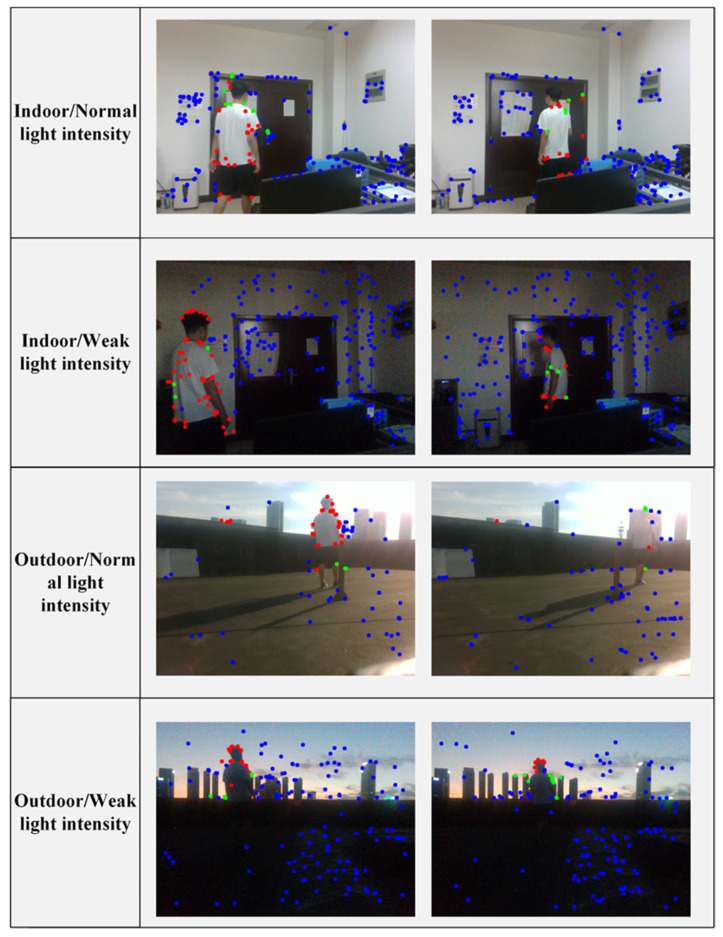
Filtering in different scenarios. The red points correspond to the dots filtered out during the first round. Green points correspond to the dots filtered in the second round.

First, we tested the filtering performance of SOLO-SLAM against dynamic points in different scenarios. The scene is divided into indoor and outdoor according to the location, and the scene is divided into normal light intensity environment and weak light intensity environment according to light intensity. Figure 14 shows the intuitive filtering effect. We can see that almost all the characteristic points on pedestrians are marked as red or green. The red dots in the figure mean the dots filtered out in the first round of filtering. The green dots in the figure mean the dots filtered out in the second round of filtering. SOLO-SLAM filters out almost all feature points in the prior dynamic region.

**Figure 15:**
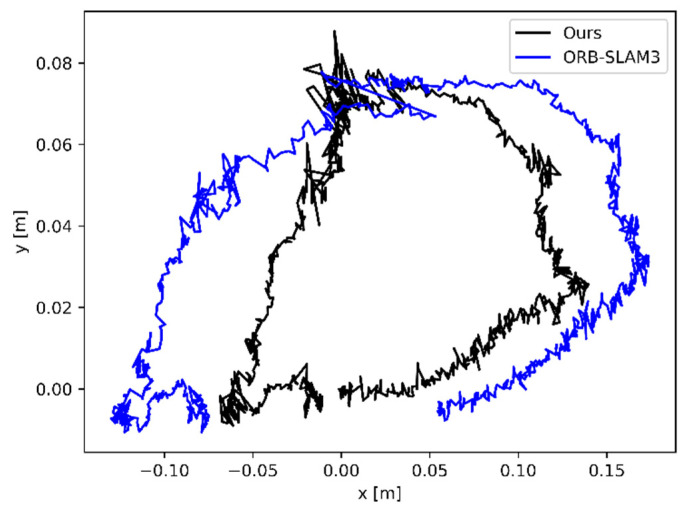
Comparison of ORB-SLAM3 and our system in indoor environment.

We also tested ORB-SLAM3 and SOLO-SLAM in the indoor environment based on the realsensed435 camera. In the test scenario, there are walking pedestrians in front of the camera. Because we could not obtain the true value of the trajectory in the real scene, we tried to make a loop motion indoors. To put this simply, we used a starting point and an ending point located at the same position as much as possible. As can be seen in Figure 15, the trajectory of SOLO-SLAM almost constitutes a loopback. In contrast, ORB-SLAM3 undergoes a significant offset.

## 5. Conclusions

In this study, we introduced a novel dynamic point filtering method based on ORB-SLAM3 with parallel processing to solve the problem of the poor real-time performance of traditional schemes as represented by Dyna-SLAM. Using the SOLO-V2 function package designed based on the ROS system, we provided semantic information for the SLAM system by combining the instance segmentation results of key frames. For semantic information, we put forward three improvements and tested them against other state-of-the-art SLAM algorithms on the TUM dynamic object dataset. The experimental validation allows us to draw the following conclusions:

1. We implemented the update of dynamic probability and semantic attributes of map points based on the instance segmentation method in a new semantic thread. Simultaneously, adopting a motion model-based tracking approach, we performed dynamic point filtering based on the dynamic state of the projected points. This approach avoids the shortcomings of the traditional sequential processing approach by reducing the processing time for obtaining the a priori dynamic regions. By comparison, our system achieves a significant improvement in real-time performance.
2. We proposed a quadratic filtering method based on the regional dynamic degree and geometric constraints to address the problem of inadequate dynamic point recognition under the parallel processing approach. By comparison, the accuracy of our system outperforms other algorithms, including the DS-SLAM scheme with sequential processing in most cases.
3. We combined the semantic attributes of map points and the semantic information of the current frame to increase the constraint term during optimization. The proposed algorithm solves the problem of accuracy degradation caused by excessive dynamic point filtering in low dynamic scenes to a certain extent. By comparison, SOLO-SLAM achieves the best accuracy in low dynamic scenes.

Although SOLO-SLAM already has good performance in dynamic scenarios, we believe that there are still possibilities for further improvements.

We note that in the low dynamic scenes in the TUM dataset, most parts of the human body are static. Based on common sense, most of the vehicles in the parking lot remain static. In the above example, directly and brutally filtering the feature points in the a priori dynamic region will lead us to lose a great deal of information that can be used. Therefore, we believe that effectively distinguishing the static parts in the a priori static region is a practical idea for future improvement.

In addition, we think it will be interesting to combine this with other sensors. In the follow-up work, we suggest improving it in combination with wheel odometry and lidar. At present, the visual SLAM algorithm is limited by the strong assumption of a static environment. When there are moving objects in the environment, the accuracy of the system will be seriously reduced due to the large number of abnormal values. In contrast, the wheel odometry obtains the motion state based on the track estimation of the wheel. This method has high locating accuracy in a short time and short distance and is not affected by dynamic targets in principle. However, the wheel odometry has some problems; for example, the accumulated error cannot be eliminated and the wheel slip introduces abnormal information. Therefore, the tight coupling of the vision method and wheel odometry method to achieve complementarity has objective and practical significance for improving system performance.

In the subsequent work, we also plan to carry out an improved solution based on the combination of vision and LIDAR. The vision SLAM and laser SLAM are similar in principle in the back-end optimization part. However, in the front-end part, LiDAR can directly provide point cloud information in the environment. These point cloud features are almost unaffected by light variations and are highly accurate. In addition to this, the point cloud information is returned directly from the laser scanner without additional computation. This also helps to achieve real-time scene building. Therefore, the combination of LiDAR helps us to further enhance the real-time mapping capability of the SLAM system and to overcome the effects of drastic illumination changes.

## Figures and Tables

**Table 1:** Experimental results of absolute trajectory error (ATE [m]).

Sequences	ORB-SLAM3		Dyna-SLAM		DS-SLAM		Detect-SLAM		RDS-SLAM		SOLO-SLAM		
	Mean	RMSE	Mean	RMSE	Mean	RMSE	Mean	RMSE	SD	RMSE	Mean	RMSE	SD
fr3_w_xyz	0.6580	0.7910	**0.0132**	**0.0150**	0.0186	0.0247	-	0.0241	0.0571	0.0571	0.0167	0.0187	0.0085
fr3_w_static	0.2648	0.2709	**0.0062**	**0.0060**	0.0073	0.0081	-	-	0.0206	0.0206	0.0093	0.0104	0.0046
fr3_w_rpy	0.5404	0.6390	**0.0242**	**0.0350**	0.3768	0.4442	-	0.2959	0.1604	0.1604	0.0894	0.1194	0.0791
fr3_w_hs	0.2957	0.3176	**0.0212**	**0.0250**	0.0258	0.0303	-	0.0514	0.0807	0.0807	0.0240	0.0276	0.0137
fr3_s_hs	0.0193	0.0239	0.0262	0.0170	0.0140	0.0157	-	0.0273	-	-	**0.0120**	**0.0135**	**0.0062**

**Table 2:** Boosting results of SOLO-SLAM relative to other algorithms.

Sequences	Improvements against ORB-SLAM3		Improvements against Dyna-SLAM		Improvements against DS-SLAM		Improvements against Detect-SLAM	
	Mean	RMSE	Mean	RMSE	Mean	RMSE	Mean	RMSE
fr3_w_xyz	97.46%	97.63%	10.22%	24.13%	-	22.24%	85.11%	67.18%
fr3_w_static	96.48%	96.16%	−27.86%	−28.40%	-	-	77.67%	49.51%
fr3_w_rpy	83.46%	81.32%	76.27%	73.12%	-	59.65%	50.69%	25.56%
fr3_w_hs	91.89%	91.30%	7.00%	8.78%	-	46.23%	83.02%	65.75%
fr3_s_hs	37.84%	43.55%	13.91%	13.74%	-	50.55%	-	-

**Table 3:** Experimental results of translational relative trajectory error.

Sequences	ORB-SLAM3		Improvements against ORB-SLAM3		Dyna-SLAM		DS-SLAM		Improvements against DS-SLAM		SOLO-SLAM	
	Mean	RMSE	Mean	RMSE	Mean	RMSE	Mean	RMSE	Mean	RMSE	Mean	RMSE
fr3_w_xyz	0.2033	0.225	**91.54%**	**91.11%**	0.0144	0.0165	0.0229	0.0339	**24.89%**	**41.00%**	0.0172	0.02
fr3_w_static	0.0264	0.02726	**60.98%**	**57.08%**	0.0078	0.007	0.0087	0.0097	−18.39%	−20.62%	0.0103	0.0117
fr3_w_rpy	0.0794	0.0994	**60.83%**	**54.53%**	0.0279	0.0363	0.0657	0.087	**52.66%**	**48.05%**	0.0311	0.0452
fr3_w_hs	0.0379	0.0512	**52.77%**	**58.20%**	0.0166	0.0192	0.0218	0.0275	**17.89%**	**22.18%**	0.0179	0.0214
fr3_s_hs	0.0139	0.0169	**0.72%**	**4.14%**	0.0247	0.0352	0.0146	0.0167	**5.48%**	**2.99%**	0.0138	0.0162

**Table 4:** Experimental results of rotational relative trajectory error.

Sequences	ORB-SLAM3		Improvements against ORB-SLAM3		Dyna-SLAM		DS-SLAM		Improvements against DS-SLAM		SOLO-SLAM	
	Mean	RMSE	Mean	RMSE	Mean	RMSE	Mean	RMSE	Mean	RMSE	Mean	RMSE
fr3_w_xyz	0.1461	0.1777	**91.99%**	**89.87%**	0.0097	0.0109	0.0132	0.019	**11.36%**	**5.26%**	0.0117	0.018
fr3_w_static	0.1344	0.159	**94.64%**	**95.03%**	0.0055	0.006	0.0064	0.0068	−12.50%	−16.18%	0.0072	0.0079
fr3_w_rpy	0.1221	0.1545	**63.47%**	**10.55%**	0.0164	0.0219	0.0501	0.0656	**10.98%**	−110.6%	0.0446	0.1382
fr3_w_hs	0.0232	0.0334	**44.40%**	**55.09%**	0.0128	0.0149	0.0159	0.0195	**18.87%**	**23.08%**	0.0129	0.015
fr3_s_hs	0.012717	0.014356	**1.71%**	**2.48%**	0.0175	0.0253	0.0167	0.0142	**25.15%**	**1.41%**	0.0125	0.014

**Table 5:** Comparison of Dyna-SLAM and SOLO-SLAM processing times.

Sequences	fr3_w_xyz	fr3_w_static	fr3_w_rpy	fr3_w_hs	fr3_s_hs
	Average processing time per frame [ms]				
Dyna-SLAM [ms]	674.68	587.60	574.66	643.86	578.68
SOLO-SLAM [ms]	67.00	67.00	67.00	67.00	67.00
Improvements against DynaSLAM	90.07%	88.60%	88.34%	89.59%	88.42%

## Data Availability

The dataset used in the paper is the public TUM dataset. It can be downloaded at this link: https://vision.in.tum.de/data/datasets/rgbd-dataset.
